# Longitudinal analysis of antibody decay in convalescent COVID-19 patients

**DOI:** 10.1038/s41598-021-96171-4

**Published:** 2021-08-18

**Authors:** Weiming Xia, Mingfei Li, Ying Wang, Lewis E. Kazis, Kim Berlo, Noureddine Melikechi, Gregory R. Chiklis

**Affiliations:** 1 Geriatric Research Education Clinical Center, Bedford VA Healthcare System, Bedford, MA 01730 USA; 2 Department of Pharmacology and Experimental Therapeutics, Boston University School of Medicine, Boston, MA USA; 3 Center for Healthcare Organization and Implementation Research, Bedford VA Healthcare System, Bedford, MA USA; 4 Department of Mathematical Sciences, Bentley University, Waltham, MA USA; 5 Department of Health Law, Policy and Management, Boston University School of Public Health, Boston, MA USA; 6 Geotop and the Department of Earth and Planetary Sciences, McGill University, Montreal, Canada; 7 Department of Physics and Applied Physics, Kennedy College of Sciences, University of Massachusetts Lowell, Lowell, MA USA; 8 MRN Diagnostics, Franklin, MA USA

**Keywords:** Immunology, Infectious diseases

## Abstract

Determining the sustainability of antibodies targeting severe acute respiratory syndrome coronavirus 2 (SARS-CoV-2) is essential for predicting immune response against the Coronavirus disease 2019 (COVID-19). To quantify the antibody decay rates among the varying levels of anti-nucleocapsid (anti-N) Immunoglobulin G (IgG) in convalescent COVID-19 patients and estimate the length of time they maintained SARS-CoV-2 specific antibodies, we have collected longitudinal blood samples from 943 patients over the course of seven months after their initial detection of SARS-CoV-2 virus by RT-PCR. Anti-N IgG levels were then quantified in these blood samples. The primary study outcome was the comparison of antibody decay rates from convalescent patients with high or low initial levels of antibodies using a mixed linear model. Additional measures include the length of time that patients maintain sustainable levels of anti-N IgG. Antibody quantification of blood samples donated by the same subject multiple times shows a gradual decrease of IgG levels to the cutoff index level of 1.4 signal/cut-off (S/C) on the Abbott Architect SARS-CoV-2 IgG test. In addition, this study shows that antibody reduction rate is dependent on initial IgG levels, and patients with initial IgG levels above 3 S/C show a significant 1.68-fold faster reduction rate compared to those with initial IgG levels below 3 S/C. For a majority of the donors naturally occurring anti-N antibodies were detected above the threshold for only four months after infection with SARS-CoV-2. This study is clinically important for the prediction of immune response capacity in COVID-19 patients.

## Introduction

One year after the initial outbreak of the severe acute respiratory syndrome coronavirus 2 (SARS-CoV-2) pandemic, we are relying on vaccines to fight this potentially fatal coronavirus disease 2019 (COVID-19). There are many clinical manifestations related to SARS-CoV-2 infection including a systemic hyperinflammation among multiple organs^1^. Seroconversion of specific immunoglobulin M (IgM) and G (IgG) antibodies occurs as early as the fourth day after symptom onset^2^, and IgM/G levels reach plateau within six days after IgM/IgG seroconversion^3^. Appearance of IgA occurs early in seroconversion and peaks after three weeks, and is more persistent than IgM^4^. In a longitudinal study following virus-specific IgG in 76 convalescent subjects for ~ 100 days, IgG decay was found in most of those subjects, while a subset of subjects with a shorter recovery period had stable antibody levels^5^.

Naturally occurring antibodies target spike protein (anti-S) and nucleoprotein (anti-N) of SARS-CoV-2 and reflect immune responses among COVID-19 patients. Antibodies from 190 control subjects enrolled during pre-COVID-19 era recognize SARS-CoV-2 open reading frame 1 (ORF1); in contrast, antibodies from 232 COVID-19 patients recognize the spike protein and nucleoprotein^6^. Among 270 PCR-confirmed COVID-19 patients, antibodies recognizing the receptor binding domain (RBD) of the viral protein have weaker responses in non-hospitalized patients, compared to those from hospitalized patients^7^. In a study of 1850 COVID-19 patients, spike protein (S)-, RBD-specific IgM and IgG levels were found to be 1.5-fold higher among severe/critical patients compared to patients with mild to moderate disease severity^8^. The RBD-specific IgG levels were found to be four-fold higher in older patients than those in younger patients^8^. Among hospitalized patients, males produced stronger SARS-CoV-2 antibody responses than females^6^. While female patients have more robust T cell activation than male patients, male patients have higher plasma levels of innate immune cytokines and a more robust induction of non-classical monocytes in blood^9^.

In a recent study, Dan et al*.* show that anti-N IgG kinetics are similar to anti-S IgG over 8 months^10^. In this study, we followed 943 patients for over 200 days after the last day showing symptoms and quantified anti-N IgG levels and their decay rate to determine the length of time patients who have recovered from SARS-CoV-2 infection retained IgG levels. We focused on a longitudinal recording of antibody responses over the course of 7 months and provided estimation of the immune response capacity of COVID-19 patients.

## Materials and methods

### Materials

Reagents used for biochemical assays and sample preparation were purchased from Thermo Scientific (Rockford, IL). The Architect SARS-CoV-2 IgG assay was obtained from Abbott (Abbott Park, IL). The SARS-CoV-2 IgG assay is a chemiluminescent microparticle immunoassay (CMIA) with 100% sensitivity and 99.6% specificity; it is intended for the qualitative detection of IgG antibodies to SARS-CoV-2 nucleocapsid protein in serum and plasma from individuals who are suspected or may have been infected by SARS-CoV-2^11^. The unit of IgG level was defined by signal/cut-off (S/C) per manufacturer’s Instruction for Use (IFU) with an assay cutoff of 1.4 (S/C).

### Subjects

Convalescent plasma was collected from subjects under the national expanded access protocol (EAP) sponsored by the Mayo Clinic. This program was established in April 2020 and has since enrolled > 100,000 subjects^12^. Blood collection was approved by institutional review committee Diagnostics Investigational Review Board. All studies were performed in accordance with FDA guidelines and Code of Federal regulations including IRB approval, and informed consent was obtained from all participants for blood donation. The collections were carried out under an Emergency Investigational New Drug (eIND) design approved by the FDA. There is no personal identifiable information, symptom level, and disease severity available for any of the donors in this study. Since the onset of the pandemic in February 2020, blood was collected from these subjects every ten days, and the data was censored as of November 2020. Over three thousand records were retrieved for this study (Supplement Table S1). COVID-19 positive/negative patients were identified by nucleic acid RT-PCR detection of SARS-CoV-2 virus using Emergency Use Authorization (EUA) approved molecular tests. Since eIND pursues convalescent plasma, COVID-19 negative subjects were not followed. COVID-19 positive patients provided blood one or more times for IgG measurement. The dates of PCR testing, the last day showing symptoms, and the dates of blood donation were recorded. Among 943 subjects, there were 471 subjects who donated blood once. A total of 472 subjects donated blood two or more times, including 214 patients whose anti-N IgG levels rebound one or more times, and 258 patients whose anti-N IgG levels monotonically decreased from the first to last dates of blood donation. We excluded seven outlier subjects (whose initial anti-N IgG level was below 0.1 S/C), as we did not find any elevation of IgG levels over time even though viral infection was confirmed by RT-PCR. We do not have the clinical information of symptom level and disease severity of these seven subjects. We have analyzed anti-N IgG levels of 251 subjects and correlated them to the time intervals between the last day showing symptoms and the day of blood donation.

### Statistical analysis

To estimate the average anti-N IgG level at each time point, we applied a mixed linear model on patients’ anti-N IgG level as the dependent variable while controlling for random effects of patients and the fixed effect of time points. The mixed linear model allows a wide variety of correlation patterns to be modeled and provides a robust approach in these situations.

The hierarchy of levels for our mixed model includes the subjects (level 2) and the multiple anti-N IgG measurements within subjects (lower level 1). The multiple IgG measurements within the same subject decreased over time. Using the mixed model, we studied the overall main effect of time after viral infection on IgG measurements. For each time point, the mixed model compares the mean of all IgG measurements for this time point with the mean of all IgG measurements at the reference time point (the last donation date after the last symptom date among all subjects). Next, the maximum likelihood method was used as an iterative method to find the best statistical estimate on the effects of time points and IgG levels at each time point. To better understand the overall decreasing pattern of IgG and predict its level at certain time points, we applied first and second order lines to the estimated IgG level at each time point from the mixed model. To prevent the inflation of a false positive rate in multiple comparisons, we used a Bonferroni correction to adjust the significance level. Based on a total of 160 time points, the adjusted significance level is 0.05/160 = 0.0003. With this adjusted significance level, we compared the p-values at each time point and reported the comparison result. We performed a two-sided *t* test to compare the IgG level at each time point with a positive antibody threshold of 1.4 S/C. SAS 9.4 and R 4.0.2 were used for our analysis.

## Results

### Collection of blood samples from COVID-19 patients

**Figure 1:**
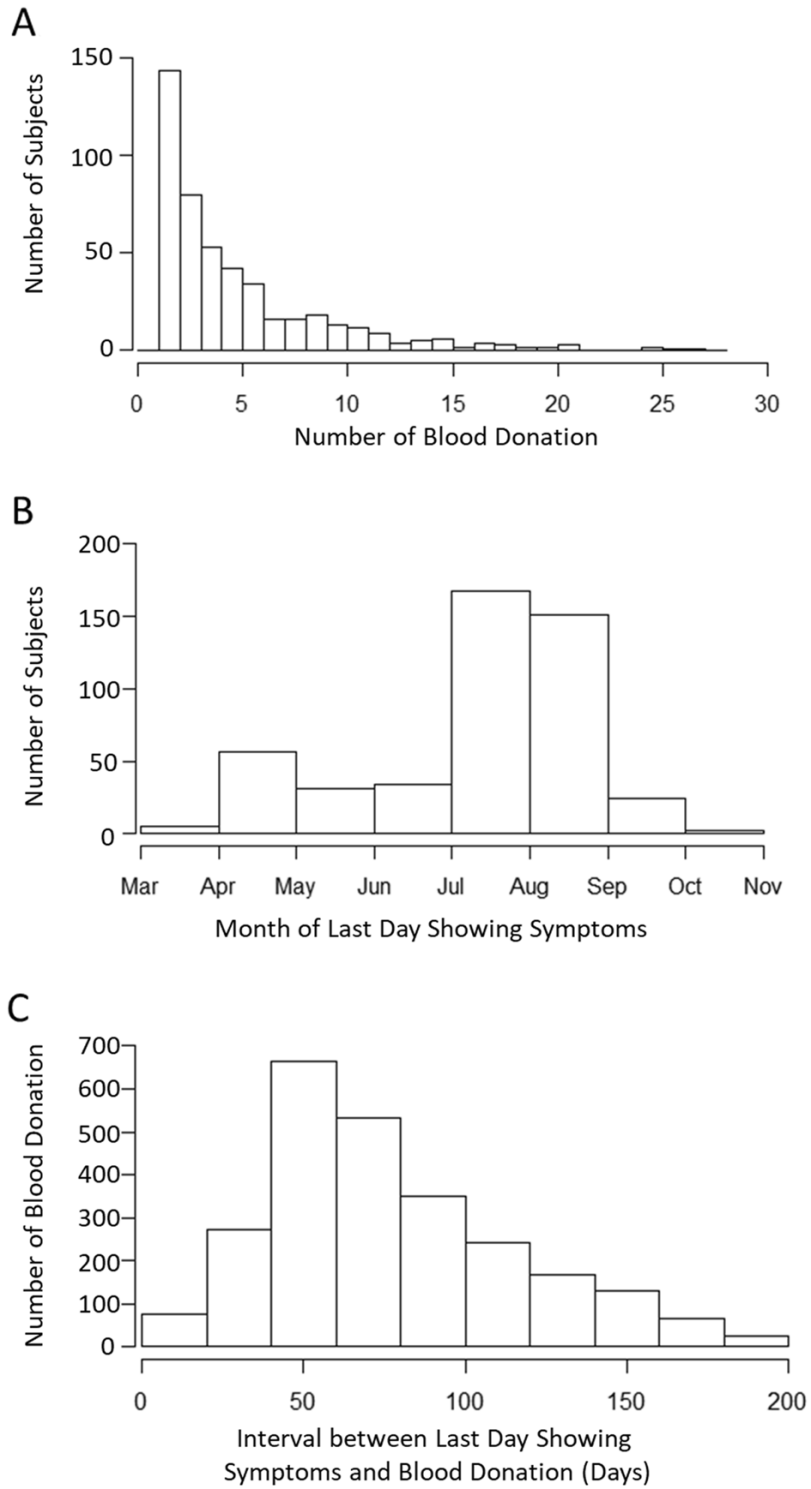
Distribution of COVID-19 patients with multiple blood donation. (**A**) Distribution of the number of blood donations from COVID-19 patients. (**B**) Distribution of patients with their last days showing COVID-19 symptoms. (**C**) Distribution of the time interval between the last day showing symptoms and the latest day of blood donation.

We have analyzed anti-N IgG levels from 943 COVID-19 patients who tested positive for SARS-CoV-2 via RT-PCR testing. Among 472 subjects with multiple donations, most people donated blood less than five times, and the maximum number of times that one person donated blood was 27 times (Fig. 1A). The majority of patients exhibited their last days of symptoms in July and August (Fig. 1B). The average time between the last day showing symptoms and the blood donation was 78 ± 38 days, up to the longest period of 200 days, which reflects timing of our blood collection and antibody test (Fig. 1C).

### Reduction of antibody levels

**Figure 2:**
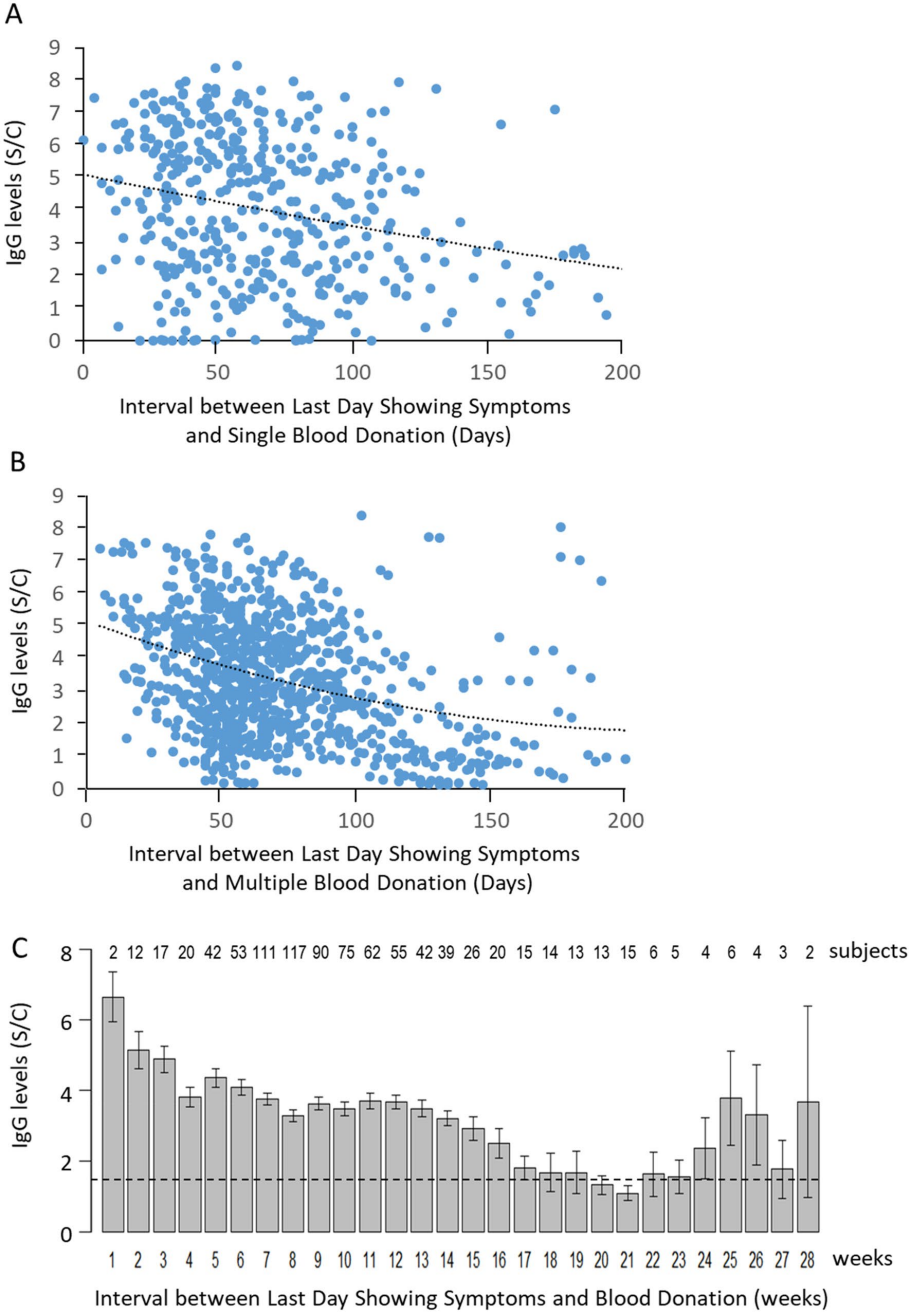
Reduction of average anti-N IgG levels in blood donated by COVID-19 positive subjects. (**A**) Anti-N IgG levels from subjects who donated blood once within 200 days after the last day showing symptoms illustrated a time-dependent decay. (**B**) All records of anti-N IgG levels in subjects who donated blood two or more times within 200 days after the last day showing symptoms are illustrated. Dotted lines represent the second order polynomial fitting curve. (**C**) The average anti-N IgG levels from blood samples donated within each week after the last day showing symptoms were calculated. The IgG levels were above 6 S/C at the first week and dropped to ~ 1.4 S/C at the 17th weeks. The dotted line represents the Abbott Architect positive/negative antibody threshold level at 1.4 S/C. Bars represent the standard error of means.

Among all subjects who donated blood once, those who donated within 100 days since the last day showing symptoms had levels of anti-N IgG reaching 9 S/C (Fig. 2A). Between 100 to 150 days, most donors had IgG levels up to 6 S/C; after 150 days, most donors carried IgG levels below 3 S/C, except for several outliers (Fig. 2A). Among 472 subjects who donated blood two or more times, 251 subjects exhibited their highest IgG levels on the first day of donation and the lowest IgG levels on the last day of donation, with time-dependent linear reduction of IgG levels (Supplement Fig. S1A,B). When IgG levels from these subjects were illustrated, we found a similar distribution of IgG levels at different intervals between the last day showing symptoms and multiple blood donation days (Fig. 2B).

We calculated the average anti-N IgG levels each week after the last day showing symptoms. We found that the average IgG level dropped from > 6 S/C to 5 S/C after the first week. This level continued to decrease to 1.4 S/C, the threshold level of the Abbott Architect positive/negative antibody test (Fig. 2C, dotted line), during the 17th week (120 days). The lowest level was measured during the 21st week (e.g., 150 days). A large variation of IgG levels was observed and attributed to a small number of subjects who donated blood after 21 weeks (Fig. 2C). The anti-N IgG levels from some of these subjects remained high for 200 days (Fig. 2B), leading to an increase in average levels of IgG among a small number of subjects (Fig. 2C).

### Antibody reduction rate is dependent on initial anti-N IgG levels

**Figure 3:**
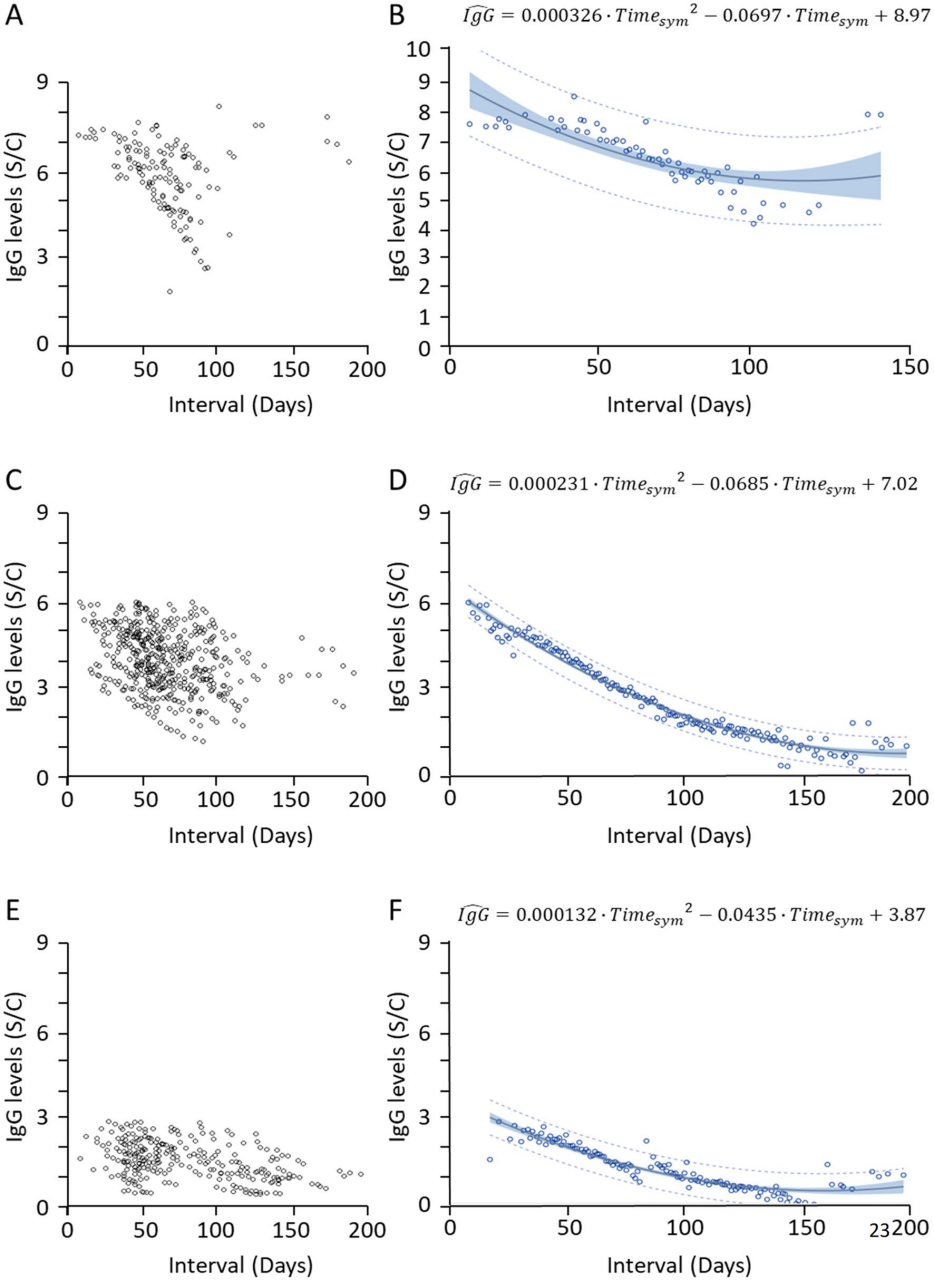
Antibody decay rates in subjects with variable anti-N IgG levels. (**A**) Distribution of anti-N IgG levels from subjects whose first donation of blood carried high levels of IgG (above 6 S/C). (**B**) Estimated mean anti-N IgG levels at each time point from the mixed model estimation. (**C**) Distribution of anti-N IgG levels from subjects whose first donation of blood carried medium levels of IgG (between 3 and 6 S/C). (**D**) Estimated mean anti-N IgG levels at each time point from the mixed model estimation. (**E**) Distribution of anti-N IgG levels from subjects whose first donation of blood carried low levels of IgG (below 3 S/C). (**F**) Estimated mean anti-N IgG levels at each time point from the mixed model estimation. Each dot represents the estimated IgG level at that time point. In (**B**, **D**, **F**) the solid curve line is the estimated fitting model. The blue shaded band shows the 95% confidence interval for the IgG level, and the dashed band shows the 95% prediction interval for the IgG level with this model.

We separated all subjects into three groups, based on the anti-N IgG levels from their first donated blood samples. We searched for any difference in antibody reduction rates in subjects carrying high vs. low levels of IgG. The first group of 36 subjects had starting IgG levels above 6 S/C; two subjects sustained high levels of IgG for more than 6 months and were excluded for the calculation of reduction rate (Fig. 3A).

We used a mixed model to estimate the reduction rate of anti-N IgG levels for these 34 subjects. The mixed model focuses on the overall effect of time after viral infection on IgG measurements. Our mixed model included the subjects and the multiple IgG measurements within subjects, which were decreasing over the time. We applied a second order polynomial line to the estimated IgG level at each time point. The estimated fitting curve is $\widehat{IgG}=0.000326\cdot {Tim{e}_{sym}}^{2}-0.0697\cdot Tim{e}_{sym}+8.97$, where Time_sym_ represents the time interval between the last day showing symptoms and the day of blood donation (Fig. 3B). We also created a first order linear fitting curve, $\widehat{IgG}=-0.0284\cdot Tim{e}_{sym}+7.92.$ When the initial IgG levels were high (> 6 S/C), the reduction rate was approximately at 0.0284 ± 0.0077 S/C per day.

A second group of 129 subjects with initial anti-N IgG levels between 3 and 6 S/C were followed for 200 days. We found that the levels of IgG were clustered within 120 days, and a few subjects maintained their high IgG levels up to 200 days (Fig. 3C). We also followed individual subject’s IgG levels and applied the same mixed model to estimate the reduction rate (Fig. 3D), using a second order polynomial fitting curve, $\widehat{IgG}=0.000231\cdot {Tim{e}_{sym}}^{2}-0.0685\cdot Tim{e}_{sym}+7.02$. The first order linear fitting curve is $\widehat{IgG}=-0.0283\cdot Tim{e}_{sym}+5.71,$ and the reduction rate was estimated to be 0.0283 ± 0.0035 S/C per day, similar to those from the first group of subjects with higher initial IgG levels.

A third group of 86 subjects had low initial anti-N IgG levels (< 3 S/C) (Fig. 3E). When individual subject’s IgG levels were followed using the same mixed model, a much slower reduction rate was observed (Fig. 3F). A second order polynomial fitting curve was used to estimate IgG level at each time point, $\widehat{IgG}=0.000132\cdot {Tim{e}_{sym}}^{2}-0.0435\cdot Tim{e}_{sym}+3.87$. The first order linear fitting curve is $\widehat{IgG}=-0.0168\cdot Tim{e}_{sym}+2.77$, and the reduction rate was estimated to be 0.0168 ± 0.0017 S/C per day. Thus, the reduction rate in subjects with initial IgG levels above 3 S/C was 1.68-fold faster than those with initial IgG levels below 3 S/C, and the difference is statistically significant (*p* < 0.05).

### Significant antibody decay four months after last day showing symptoms

**Figure 4:**
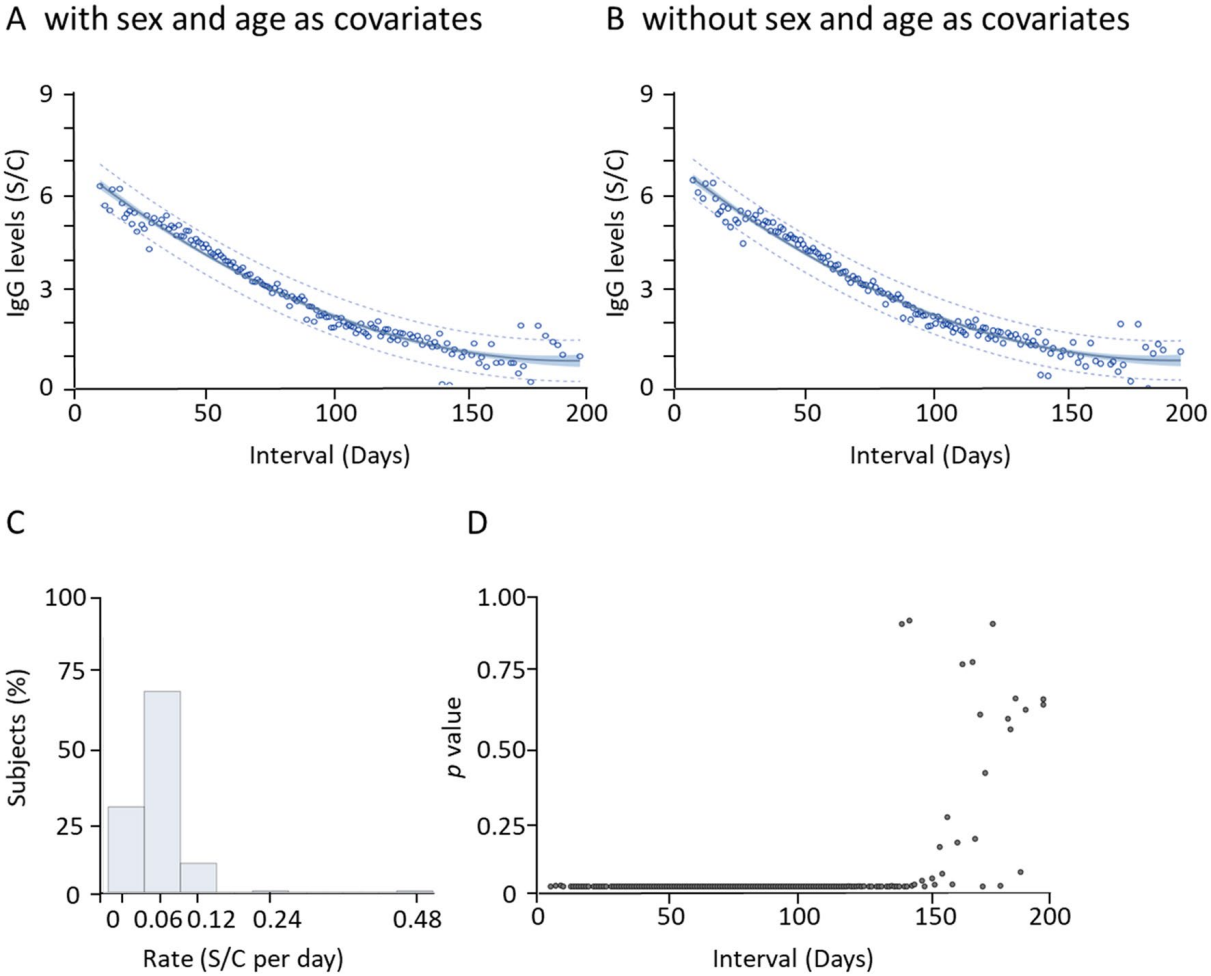
Antibody decay for 4 months after last day showing symptoms. (**A**) Mean anti-N IgG levels were calculated at each time point from the mixed model estimation with sex and age as covariates. Each dot represents the estimated IgG level at that time point. The solid curve line is the estimated fitting model. The blue shaded band shows the 95% confidence interval for the IgG level, and the dashed band shows the 95% prediction interval for the IgG level with this model. (**B**) Mean anti-N IgG levels were calculated at each time point from the mixed model estimation without sex and age as covariates. (**C**) Distribution of overall decline rates from all patients. The rate was defined as the difference between the first- and last-day anti-N IgG levels divided by the time interval. (**D**) The variation of anti-N IgG levels at individual time points from day five to day 200 since the last day showing symptoms was calculated. Due to the multiple simultaneous comparisons, Bonferroni adjustment was used to plot the *p* values against time intervals, and high variation of IgG levels was observed after 120 days.

We analyzed the overall anti-N IgG reduction across 200 days. We created a fitting curve to estimate the relative levels of IgG for all subjects using the mixed linear model with age and sex as covariates (Fig. 4A). The second order polynomial fitting curve is $\widehat{IgG}=0.000156\cdot {Tim{e}_{sym}}^{2}-0.0613\cdot Tim{e}_{sym}+6.62$. Depending on the length of time following the last day showing symptoms, most patients exhibited a gradual reduction of antibody levels. A mixed linear model without age and sex as covariates is also given (Fig. 4B). The second order polynomial fitting curve is $\widehat{IgG}=0.000156\cdot {Tim{e}_{sym}}^{2}-0.0614\cdot Tim{e}_{sym}+6.68$. We find that all patients (except for 6 outlier records; R^2^ = 0.9674) (Fig. 4B) fall within a 95% confidence interval for the fitted curve. These outcomes, without age and sex as covariates, were almost identical to those with age and sex as covariates. The distribution of reduction rates was found clustered below 0.12 S/C per day, with a few cases showing a high reduction rate at 0.48 S/C per day (Fig. 4C). Using linear curve fitting, the average decay rate for all subjects was estimated at 0.03 S/C per day (Supplement Fig. S2).

We calculated the variation of anti-N IgG levels at individual time points from day five to day 200 since the last day showing symptoms. We used the Bonferroni adjustment for multiple simultaneous comparisons and plotted the *p* values against time intervals (Fig. 4D). All patients who donated blood within 120 days (17 weeks) after their last day showing symptoms exhibited average IgG levels higher than 1.4 S/C (Fig. 2C). After 120 days, the variation of IgG levels was high (Fig. 4D). We found that the mean IgG level (± SD) for day 122 was 1.65 ± 0.26 S/C and for day 123, it was 1.32 ± 0.40 S/C. This suggests that once the IgG level reduced to 1.4 S/C, there was a large variation of IgG levels among those patients who donated their blood samples 120 or more days after the last day showing symptoms.

## Discussion

Since the start of the SARS-CoV-2 pandemic, over 187 million COVID-19 patients worldwide have recovered from the infection, and convergent antibody responses to SARS-CoV-2 in convalescent individuals have been intensely investigated for therapeutic applications^13^. We have followed our convalescent subjects for over 200 days, and our work represents one of the first reported longitudinal studies over the course of a half year^10,14^. Our study yields unique information on variable antibody decay rates among different convalescent patients.

On average, antibodies reach constant levels by 16 to 30 days post symptom onset (PSO)^5,15^. Based on our calculation, the rate of anti-N IgG reduction was relatively higher at the beginning, then gradually decreased to a steady state after four months (Fig. 4), at which point the fluctuation of IgG levels exhibited random variation across all recovered patients. This is consistent with a previous report that anti-RBD-IgG responses decayed slowly through 90 days in 343 COVID-19 patients out of a total of 122 days under investigation^16^. The anti-RBD-IgG antibodies are correlated with anti-S neutralizing antibody titers that remain steady for 75 days PSO^16^. In a separate study of samples collected up to 115 days PSO, IgG levels were found to be stable for up to three months^15^, which is longer than a 49-day half-life found in a cohort of 647 SARS-CoV-2 infected patients^17^. This suggests that the antibodies generated from initial SARS-CoV-2 infection were sustained for a significant period of time and primed the adaptive immune response with B and T cells, which may prevent severe COVID-19 outcomes^18–20^.

Responses of specific T cells to the viral spike protein are correlated with anti-SARS-CoV-2 IgG and IgA titers^21^. On the other hand, a strong cytotoxic Tfh response is correlated negatively with anti-S antibody levels^22,23^. Anti-S antibodies, targeting the RBD, N-terminal domain (NTD) and a third region that bridges two separate RBDs^24^, could block the binding of ACE2^25^, with highly potent 50% virus-inhibitory concentrations in the range of 0.7–15 ng/ml^13,24,26^. Similar to findings in the UK Biobank SARS-CoV-2 Serology Study, the latest findings from convalescent donors illustrate the retention of low levels of IgG antibodies in 80% of blood samples collected at 6–8 months after symptom onset, with the persistent presence of SARS-CoV-2-specific memory B- and T-cells^10,14^.

Sustainability of antibodies after infection varies across populations. We have identified a few subjects whose anti-N IgG levels were sustained at high levels for about 200 days (Fig. 3A). These subjects donated blood multiple times with little reduction of IgG levels. Further examination of these subjects will help us understand variations in acute infection, convalescence, and memory phases from SARS-CoV-2^27^. The Abbott Architect anti-N IgG assay has been widely used to measure IgG levels in the population. Use of this assay for our COVID-19 patients revealed an excellent agreement between our patients’ IgG levels at a time point around day 120 and the threshold level of 1.4 S/C, established by the Abbott IgG assay for the positive antibody presence. We found that our patients’ IgG levels dropped below 1.4 S/C and became random after 120 days post the last day showing symptoms. It will be important to compare our findings to anti-S IgG in future studies among vaccinated subjects.

Our studies have several limitations. First, we did not measure neutralizing antibody titers of our samples. An understanding of enhancement and decaying of neutralizing antibody titers will provide the functional association with the course of viral infection and immune responses. Second, we did not separate age and sex as independent covariates for our analysis, even though we found similar outcomes when age and sex were either included or excluded as covariates. It is clear that aging plays an important role in responses to COVID-19^28^; older people have a reduced amount of CD4+ and CD8+ T cells, and poor T cell response was found negatively correlated with the age of patients^9,29^. Sex differences exist in immune responses with female subjects having more T cell activation with better disease outcomes compared with male patients^9^. Third, we only used the Abbott Architect assay for IgG against nucleocapsid protein, therefore, other antibody tests may detect different IgG levels after 120 days. It was reported that IgG against the spike protein was stable over 6 months^10^. It is important to rule out that our 120-day time line is not a function of the specific assay used in this study. While the anti-N IgG levels of convalescent plasma samples have been used as an indicator of immune response among COVID-19 patients, the levels of anti-S or anti-RBD antibodies are likely to be more informative for predicting protective potential. Based on a systematic review of ~ 100 clinical studies and in vitro tests, convalescent plasma therapy has demonstrated efficacy in saving patients who are critically ill or mechanically ventilated and resistant to antivirals and supportive care^30^. Therefore, evaluating IgG levels in convalescent plasma samples is clinically important.

In summary, we have demonstrated that naturally occurring antibodies decreased at different rates during the four months after infection with SARS-CoV-2. It is clinically important to predict the immune response capacity and to prevent COVID-19 re-infection. Future studies are needed to understand the sustainability of antibodies and time course of eliciting the adaptive immune response both in an initial exposure to SARS-CoV-2 virus and after vaccination.

## Supplementary Information

Supplementary Information.
