# Supplementary material for: Longitudinal analysis of antibody decay in convalescent COVID-19 patients
Source: Sci Rep. 2021 Aug 18;11:16796. doi: 10.1038/s41598-021-96171-4 (PMC8373894; doi:10.1038/s41598-021-96171-4)
Supplement: Supplementary file 1 — Supplementary Information. [file 41598_2021_96171_MOESM1_ESM.docx]

**Supplement Table S1**

**Table S1 Characteristics of convalescent COVID-19 patients**

| Sample Size | Number of records | 3,024 |
| --- | --- | --- |
|  | Number of records excluding  Interval > 1 year | 2,998 |
|  | Number of donors | 943 |
|  | Number of donors excluding interval >1 year | 934 |
| Age | Min | 17 |
|  | Max | 94 |
|  | NA | 17 |
|  | Mean | 41.7 |
|  | SD | 13.3 |
| Sex | Female | 433 |
|  | Male | 484 |
|  | NA | 17 |
| Interval days between the last symptom and the first donation | Min | 0 |
|  | Max | 193 |
|  | NA | 17 |
|  | Mean | 57.4 |
|  | SD | 34.3 |
| PCR test date | Earliest | 2/21/2020 |
|  | Latest | 10/5/2020 |
|  | NA | 77 |
| PCR results | Positive | 873 |
|  | Negative | 29 |
|  | NA | 32 |

NA: record not available; SD: standard deviation.

**Supplement Figures**

**Figure Legend**

**Figure S1 Reduction of anti-N IgG levels in two COVID-19 positive individuals**.

**A**. Anti-N IgG levels from subject A who donated blood 16 times within 100 days after the last day showing symptoms illustrated a time-dependent decay. **B**. Anti-N IgG levels from subject B who donated blood 11 times within 100 days after the last day showing symptoms illustrated a similar time-dependent decay.

**Figure S2 Time-dependent, first order linear reduction of antibodies after last day showing symptoms**.

Mean anti-N IgG levels were calculated at each time point from the mixed model estimation. Each dot represents the estimated IgG level at that time point. The solid line uses the first order linear fitting model. The dashed band shows the 95% prediction interval for the IgG level with this model.


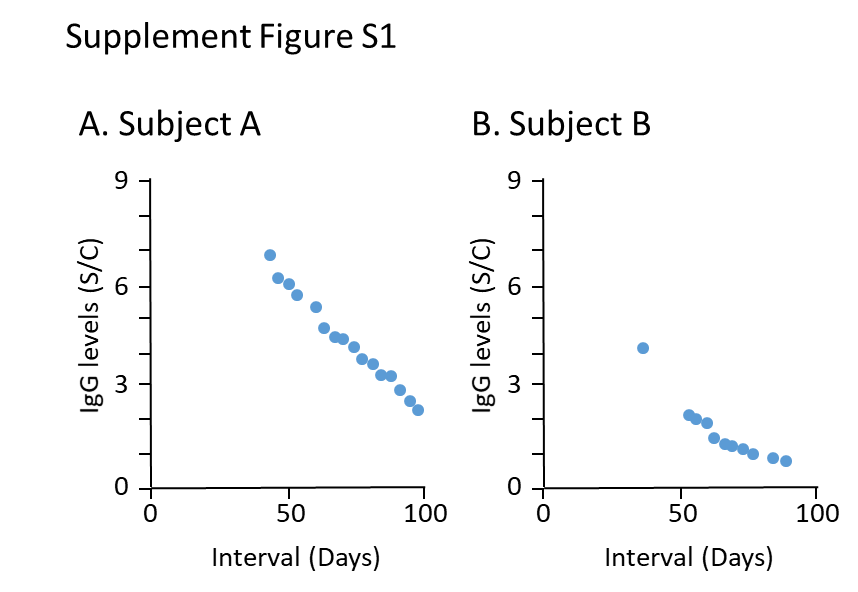


**
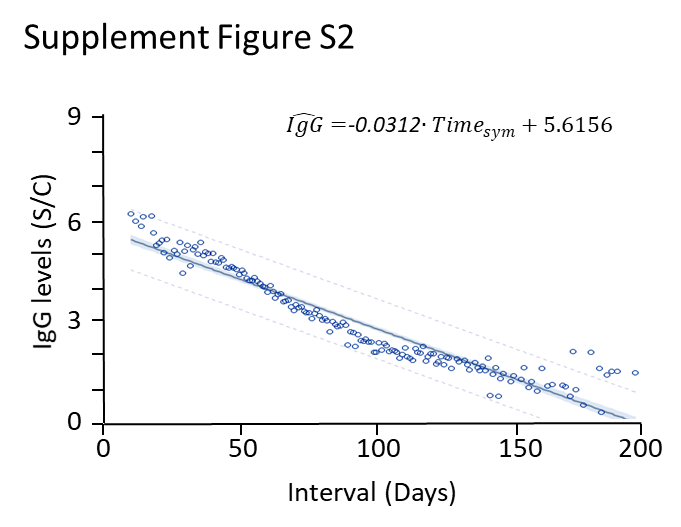
**
